# Regional Differences of Guillain-Barré Syndrome in China: From South to North

**DOI:** 10.3389/fnagi.2022.831890

**Published:** 2022-02-01

**Authors:** Jiajia Yao, Yin Liu, Shuping Liu, Zuneng Lu

**Affiliations:** Department of Neurology, Renmin Hospital of Wuhan University, Wuhan, China

**Keywords:** Guillain-Barré syndrome (GBS), China, epidemiological, regional difference, AIDP (acute inflammatory demyelinating polyneuropathy), AMAN (acute motor axonal neuropathy)

## Abstract

**Background:**

The epidemiological features of Guillain-Barré syndrome (GBS) were different in different areas; a comparison of the disease was needed to identify the variation and prognosis. We compare the epidemiological features of GBS in different areas in China.

**Method:**

A total of 1,191 patients were included. Information was collected in patients diagnosed with GBS and its variants in middle and south China, and then retrospectively reviewed. The patients were divided into four different regions: East China (*n* = 441), Center China (*n* = 566), South China (*n* = 77), and Southwest China (*n* = 107). These subregions are mainly divided by climate and geographical location. These data were compared with data from a study in East China (Shandong, *n* = 150) and Northeast China (Changchun, *n* = 750).

**Results:**

Patients from the south and southwest China were younger than other regions (*P* = 0.000). A summer peak and an autumn peak were found in northern China, but more patients in winter and spring days in other areas (*P* = 0.000). Upper respiratory tract infection (URTI) was the preceding event of GBS patients in all regions but rarer in central China (*P* = 0.001). The proportion of axonal subtype was higher in central and southwest China than in other regions (*P* = 0.001). Patients in southwest China were more served at nadir and have the longest hospital stay (*P* = 0.003 and *P* = 0.000).

**Conclusion:**

The difference between seasonal variation and preceding events was found in different regions in China; clinical features differ among regions in China.

## Introduction

Guillain-Barré syndrome (GBS) is an acute polyradiculoneuropathy, presenting with muscle weakness and sensory symptoms; regional differences were found worldwide (Hughes and Cornblath, 2005; Greenberg and Vearrier, 2012; Kuwabara and Yuki, 2013; Wakerley et al., 2014; Shahrizaila et al., 2021). A study about the regional differences in Europe and Asia on GBS in 2018 yielded positive results, but the study did not include China (Doets et al., 2018). China has a vast territory, and the climate and economic development levels vary from region to region; it is unknown whether it varies in different regions of China. Our study has found that demyelinating was the major subtype in south China with a percentage of 49%, different from previous studies in north China (Ho et al., 1995; Liu et al., 2018).

Comparisons of previous studies suggest that the geographical origin of patients with GBS influences the variations of this disease, such as axonal, was the main subtype in Northern China (Ho et al., 1995), and demyelinating was more common in South China, European, and American countries (Hadden et al., 1998; Hughes and Cornblath, 2005; van Doorn et al., 2008; Liu et al., 2018); Europe shows an increased incidence in winter or spring, while India and Latin America receive a significant decrease in winter (Webb et al., 2015). Studying the characteristics of GBS in different regions can understand the severity and subtypes of diseases caused by environmental or population differences so as to provide more accurate solutions for disease diagnosis and treatment. Regional variation of GBS needs to be carefully discussed.

Eating habits vary in different regions in China. Climate also varies between south and north China; northern China has more distinct seasons, while the south has more rainy days. The medical level and the economic level vary in different regions in China; poor medical resources may lead to misdiagnosis or delayed treatment, and regional differences in different provinces in south China need further analysis to help make suitable decisions for local patients.

## Materials and Methods

### Study Population

Patients diagnosed with GBS and its variants were collected from January 2013 to September 2016 in several representative hospitals of equal standard in 14 provinces in middle and south China (Hospitals are listed in Supplementary Table 1). Neurologists in the Department of Neurology, Renmin Hospital of Wuhan University, and well-trained GBS team members help validate the diagnosis of each patient. Classical GBS confirms the criteria of Asbury and Cornblath (1990); Miller-Fisher syndrome (MFS) was diagnosed according to the new diagnostic classification (Wakerley et al., 2014). Subtypes of patients with GBS were classified according to the criteria, which were wildly accepted (Ho et al., 1995; Hadden et al., 1998; Hughes and Cornblath, 2005). Admissions were divided into four seasons (spring: March-May, summer: June-August, autumn: September-November, and winter: December-February). Patients who abandoned the examination or treatment within 5 days after the admission, and those who could not rule out other causes of the weakness or relapsed were excluded. Data from other provinces in China were carefully collected from PubMed by searching the keywords “Guillain-Barré syndrome,” “epidemiological,” and “region.” Parts of research that meet the same electrodiagnostic criteria and disease severity evaluation were enrolled in our study for further comparison.

### Data Collection

Information, such as age, sex, season, place of residence, preceding events, findings of electrodiagnosis, and other findings of laboratory and physical examinations, were collected by team members. All the clinical information and nerve conduction study findings were carefully checked by well-trained team members to ensure that the patients matched the diagnosis of GBS. All information was extracted on paper first and then checked and typed into the computer for further analysis.

### Geographical Regions

**FIGURE 1:**
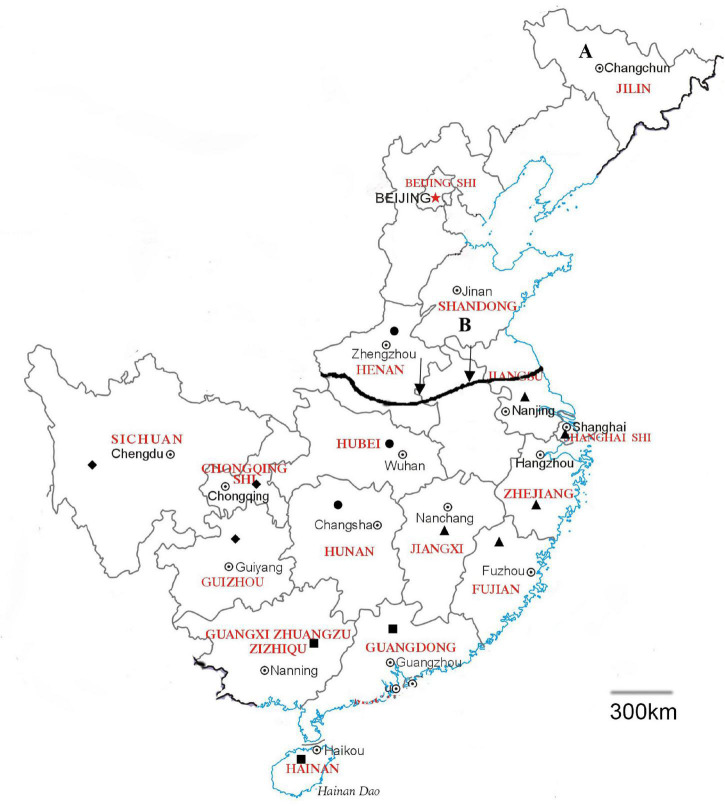
Geographical location of the regions in China. East China including Shanghai, Jiangsu, Zhejiang, Fujian, Jiangxi (▲), Central China including Hubei, Hunan, Henan (●), South China including Guangdong, Guangxi, Hainan (■), and Southwest China including Sichuan, Chongqing, Guizhou (◆). The arrow shows the Qinling-Huaihe line as the dividing line of Southern and Northern China. A: Jilin. B: Shandong. A scale of 300 km was shown in the map.

To study the influence of geographical differences on GBS heterogeneity, we divided the patients into four different regions: “East China” (including Shanghai, Jiangsu, Zhejiang, Fujian, Jiangxi), “Central China” (including Hubei, Hunan, Henan), “South China” (including Guangdong, Guangxi, Hainan), and “Southwest China” (including Sichuan, Chongqing, Guizhou). These subregions are mainly divided by climate and geographical location (Figure 1). Southern and Northern China is divided by Qinling-Huaiheline; southern China mentioned below means provinces located in the south of the dividing line of south and north, and south cities include Hubei, Hunan, Guangdong, Guangxi, Hainan, Shanghai, Jiangsu, Zhejiang, Fujian, Jiangxi, Chongqing, Sichuan, and Guizhou.

### Statistical Analysis

All statistical analysis was performed using IBM SPSS V.26.0. Categorical variables were evaluated using the χ2 test, and continuous variables were described as means and SDs, using Student’s *t*-test for normal distribution and the non-parametric test for skewed distribution. A two-sided value *p* < 0.05 was considered to be significant.

## Results

A total of 1,191 patients diagnosed with GBS were included, originated from Henan (*n* = 289), Hubei (*n* = 212), Hunan (*n* = 65), Jiangsu (*n* = 56), Shanghai (*n* = 30), Zhejiang (*n* = 289), Fujian (*n* = 41), Jiangxi (*n* = 25), Guangxi (*n* = 26), Guangdong (*n* = 31), Hainan (*n* = 20), Sichuan (*n* = 53), Chongqing (*n* = 15), and Guizhou (*n* = 39).

### Clinical Features

**FIGURE 2:**
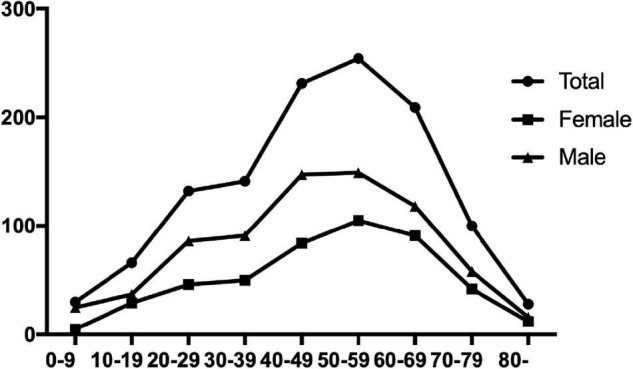
Age distribution of the patients with Guillain-Barré syndrome (GBS) in China.

The age of the patients with GBS varied from 1 to 88 years, with a median age of 49; the number of patients increased with age and reached its peak at 50–59 years. About 61% of the patients were men, and men predominated in all age categories (Figure 2).

Preceding events were found in 634 (53%) patients within 4 weeks before admission, mainly upper respiratory tract infection (61%) and gastroenteritis (16%).

About 653 (55%) patients were unable to walk independently at admission; 22 people need mechanical ventilation (Table 1).

**TABLE 1:** Demographics and clinical features of patients with GBS in China.

Age, years (IQR)	49(35–61)
Male: female ratio	727/464(1.6)
Preceding events (%)	634 (56)
URTI	387(61)
Gastroenteritis	100(16)
URTI and gastroenteritis	12(2)
Others	135(21)
**GBS disable score at admission (%)**	
Healthy,0	20(2)
Minor symptoms but able to run,1	121(12)
Able to walk independently, unable to run,2	261(25)
Not able to walk independently for at least 10m,3	265(26)
Bedridden or wheelchair bound,4	348(34)
Mechanically ventilated for at least part of the day	22(2)
**Variants of GBS (%)**	
MFS	107(69)
MFS- GBS overlap	9(6)
CNV	38(25)

*URTI: upper respiratory tract infection. MFS: Miller-Fisher syndrome. GBS: Guillain-Barré syndrome. CNV: cranial nerve variant.*

A total of 154 patients were found to be variant subtypes of GBS, including 38 (25%) CNV, 107 (69%) MFS, and 9 (6%) MFS-GBS overlap syndrome. About 750 of the remaining patients provided the available nerve conduction studies and can be classified as demyelinating (*n* = 310, 41%), axonal (*n* = 167, 22%), inexcitable (*n* = 6, 0%), equivocal (*n* = 135, 18%), and normal (*n* = 132, 18%). Compared with the demyelinating subtype, the patients with axonal GBS were a little younger (53 years versus 48 years, *P* = 0.038) and had more severe limb weakness at admission according to the GBS disable score (*P* = 0.41).

### Geographical Variation of Guillain-Barré Syndrome

The demography, seasonal variation, preceding events, clinical variants, electrophysiological subtypes, and limb weakness were compared between East China (*n* = 441), Center China (*n* = 566), South China (*n* = 77), and Southwest China (*n* = 107) (Table 2).

**TABLE 2:** Differences in GBS between regions.

	Regions of China				
	East (*n* = 441)	South (*n* = 77)	Center (*n* = 566)	Southwest (*n* = 107)	P value
Age years (IQR)	51(38–63)	41(29–52)	50(37–61)	40(16–59)	0.000
Male (%)	279(63)	44(57)	341(60)	63(59)	0.662
Season (%)	338	60	426	97	
Winter-spring	203(60)	39(65)	188(44)	55(57)	0.000
Summer-autumn	135(40)	21(35)	238(56)	42(43)	
Variants of GBS(%)	387	55	497	98	
MFS	36(67)	16(73)	52(75)	3(33)	0.072
MFS-GBS overlap	7(13)	0(0)	0(0)	2(22)	0.002
CNV	11(20)	6(27)	17(25)	4(44)	0.473
GBS disability score at admission (%)	387	55	497	98	
≥ 3	220(57)	33(60)	315(63)	66(67)	0.129
<3	167(43)	22(40)	182(37)	32(33)	
Electrophysiology subtype (%)	301	34	337	78	
Demyelinating	109(36)	13(38)	158(47)	30(38)	0.047
Axonal	49(16)	6(17)	83(25)	29(37)	0.001
Inexcitable	1(0)	1(3)	4(1)	0(0)	0.255
Equivocal	63(21)	8(24)	52(15)	12(15)	0.228
Normal	79(26)	6(18)	40(12)	7(9)	0.000
GBS disability score at nadir (%)	367	46	433	84	
≥ 3	238(65)	32(70)	323(75)	68(81)	0.003
<3	129(35)	15(30)	110(25)	16(19)	
Hospital stay days (IQR)	12(9–19)	13(10–18)	15(11–20)	17(12–22)	0.000

*MFS: Miller-Fisher syndrome. GBS: Guillain-Barré syndrome. CNV: cranial nerve variant.*

Patients from south and Southwest China were significantly younger (age, 41 years; IQR, 29–52; and age 40 years; IQR, 16–59, *P* = 0.000) than the other regions.

**FIGURE 3:**
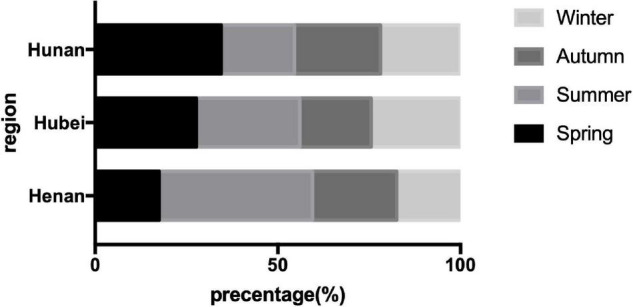
Seasonal variation of the patients with Guillain-Barré syndrome (GBS) in center China.

In Center China, we found a peak in summer and autumn days, while other regions got more patients in winter and spring days (*P* = 0.000). Seasonal variation was found in Henan province with a summer peak (*P* = 0.000), different from all the other provinces in our study (Figure 3).

**FIGURE 4:**
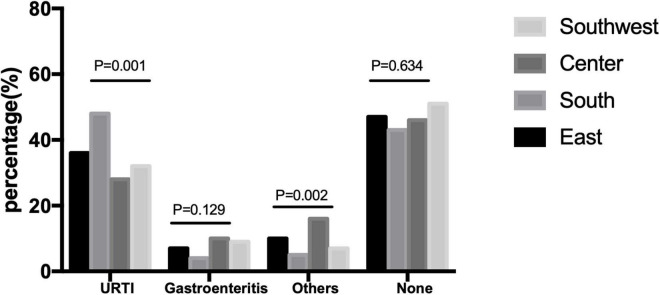
Different preceding events in different regions in China.

Upper respiratory tract infection (URTI) was the main preceding event in all regions and was rarer in Center China than in other regions (*P* = 0.001) (Table 3); no difference was found in gastroenteritis. Demyelinating was the main subtype in all regions; the proportion of axonal subtype was slightly higher in center and Southwest China than in other regions (Figure 4) (*P* = 0.001).

**TABLE 3:** Differences in the patients with GBS among different preceding events.

	URTI (*n* = 387)	Gastroenteritis (*n* = 100)	None (*n* = 557)	P value
Age years, (IQR)	46(30–58.5)	45(28–55)	52(40–63)	0.000
Male (%)	214(55)	73(73)	345(62)	0.003
**Season (%)**				
Winter-spring	166(56)	35(44)	229(52)	0.178
Summer-autumn	131(44)	44(55)	208(48)	
**GBS variant (%)**				
MFS	37(10)	4(4)	64(12)	0.066
CNV	7(4)	2(2)	16(3)	0.426
**Electrophysiology subtype (%)**				
Demyelinating	92(37)	17(24)	161(42)	0.001
Axonal	47(19)	29(41)	70(21)	0.000
Inexcitable	0(0)	0(0)	3(1)	0.163
Equivocal	39(16)	19(27)	59(17)	0.085
Normal	69(28)	8(11)	48(14)	0.000
**GBS disability score at nadir (%)**				
≥ 3	207	63	294	0.539
< 3	94	21	123	
Hospital stay days, (IQR)	14(13–19)	13(11–18)	14(10–20)	0.785

*URTI: upper respiratory tract infection. MFS: Miller-Fisher syndrome. GBS: Guillain-Barré syndrome. CNV: cranial nerve variant.*

No difference was found in the GBS disability score at admission in four regions, but the patients in Southwest China got a higher GBS disability score at nadir and longer hospital stay (*P* = 0.003 and *P* = 0.000).

The patients with URTI were more likely to develop into a demyelinating subtype of GBS (*P* = 0.001), and gastroenteritis as a preceding event was more frequently to develop into an axonal subtype. No seasonal variation was found in different preceding events, but gastroenteritis occurred more in summer and autumn. Different subtypes of GBS showed no difference in seasonal distribution (*P* = 0.156, see Supplementary Table 2).

### Comparison of Southern and Northern China

We found pieces of research on Shandong and Jilin that can match our study of Southern China; the location of these two provinces is shown in Figure 1.

The differences of the patients with GBS in southern and northern China in clinical features include a preceding event, subtype, seasonal distribution, and the GBS disability score at nadir (Table 4). The UTRI was more frequent in the southern area (*P* = 0.017), but gastroenteritis was more common in the northern area (*P* = 0.026). Demyelinating was the main subtype in both south and north; the proportion of the axonal subtype was lower in northern China (*P* = 0.008). Seasonal distribution was different in southern and northern China; we found more patients in summer and autumn days in the north (*P* = 0.000).

**TABLE 4:** Comparison of southern and northern China.

	Southern (*n* = 902)	Shandong^△^ (*n* = 150)	Jilin^▲^ (*n* = 750)	P value
Male (n,%)	555(62)	81(54)	331(62)	0.208
**Preceding event (%)**				
URTI	305(34)	36(24)		0.017^a^
Gastroenteritis	66(7)	19(13)		0.026^a^
**Electrophysiology subtype (%)**				
Demyelinating	211(39)	41(34)	92(32)	0.106
Axonal	115(21)	35(29)	45(16)	0.008
**GBS disability score at nadir (%)**				
≥ 3	535(64)	84(55)		0.065^b^
**Seasonal distribution**				
Winter-Spring	411(58)		215(40)	0.000^a^
Summer-Autumn	298(42)		326(60)	

*^△^Hao et al. (2019).*

*^▲^Wu et al. (2016).*

*^a^Comparison between our data and Changchun province.*

*^b^Comparison between our data and Shandong province.*

*URTI: upper respiratory tract infection. GBS: Guillain-Barré syndrome.*

## Discussion

Our study found that GBS has differences in electrophysiological subtypes, severity, and seasonal distribution in different regions of China.

In all regions in China, the number of patients with GBS increased with age and reached its peak at the age of 50–59 years in both men and women. A similar conclusion was found in other regions worldwide (McGrogan et al., 2009; Sejvar et al., 2011; Sipilä et al., 2017; Doets et al., 2018). Patients in South and Southwest China were a little younger than other regions; a previous study in Chongqing in Southwest China showed a median age of 47 years, similar to our conclusion (Zhang et al., 2015). The different age distributions in different regions may be due to demographic differences or different economic levels. Men were at higher risk than women with a ratio of 1.6:1, similar to previous studies worldwide (Markoula et al., 2007; Chen et al., 2014; Doets et al., 2018). Longer hospital stays and a higher GBS disability score at nadir were found in Southwest China than other regions; we thought it could be attributed to difference in economic level, and southwest of China has a lower economic level than other parts of China, especially Eastern areas. A poor economic level is reduced to poor medical conditions; it will take a longer time for disease diagnosis and treatment.

Seasonal variation was significantly different between regions; a higher summer-autumn proportion was found in Center China, especially in Henan province. Further analysis found a significant summer and autumn peak in northern China. In other regions in the world, England showed a higher incidence rate in winter, while, in northwest Greece, they found more patients in spring (Markoula et al., 2007; Webb et al., 2015). Previous studies in Northern China showed a peak of patient number in summer and autumn, but, in Jiangsu province in Southern China, there was no significant difference in patients in different seasons (Ho et al., 1995; Chen et al., 2014). Studies in north China and Bangladesh showed the late summer peak may be associated with *Campylobacter jejuni* infection (Ho et al., 1995; Islam et al., 2010); in England, the high incident rate in winter was related to influenza outbreaks (Webb et al., 2015). Northern China has a more distinct season; high temperature and precipitation increased in summer, leading to outbreaks of infection, which may be the cause of summer peak. The proportion of patients in summer and autumn gradually decreased from north to south, which indicates a changing disease spectrum in China among regions.

Demyelinating was the predominant subtype in all regions, accounting for more than 30%, which was still lower than that in Europe and North America (Hadden et al., 1998; van den Berg et al., 2014). In Southwest China, the patients have the highest proportion of an axonal subtype compared with other regions. The patients in northern China, such as Shandong and Changsha, show a proportion of the axonal subtype of 29 and 16%. A study in the 1990s in northern China showed a proportion of approximately 65% of the axonal subtype, totally different from what we found (Ho et al., 1995). Recent studies in Northeast China have found that the proportion of the axonal subtype has decreased into 25–33% (Ye et al., 2013; Zhang et al., 2017). Other Asian counties, such as Pakistan and Israel, got a proportion of more than 30 percent of the axonal type, different from those in Western countries (Shafqat et al., 2006; Kushnir et al., 2008). Studies in Bangladesh and Asia also found that demyelinating was the main subtype of GBS, but the axonal subtype was still higher in Bangladesh (Doets et al., 2018). The patients of the axonal subtype were younger than the demyelinating subtype in all regions and have a higher proportion of inability to walk independently; further analysis of other prognostic factors is needed to find the association with GBS subtypes and an outcome. These changes may relate to the changes in a disease spectrum over time. People in the rural area in China in the late 20th century usually use unboiled water in the river with animal wastes in it; poultry was also free-ranged, no hygiene habit of washing hands before meals. Urbanization has improved living standards and hygiene awareness of the people; we have little chance of contacting with polluted water. Another possible reason is that *C. jejuni* infection was not always related to the axonal type of GBS; it may perform like a demyelinating subtype either (Bae et al., 2014; Kim et al., 2014).

The URTI was the predominant preceding event of GBS in China in all regions, and the proportion of URTI in the demyelinating subtype was significantly higher than gastroenteritis. Studies about preceding infection found that cytomegalovirus and Epstein-Barr virus infection were associated with the demyelinating subtype (Sekiguchi et al., 2012). The *C. jejuni* infection is the main cause of gastroenteritis, relating to the cross-reactive antibodies to the gangliosides in the axonal subtype, and was more frequent in younger patients with GBS (Ho et al., 1995; Kuwabara and Yuki, 2013; Hao et al., 2019). A new study in China found that Influenza B was more frequently related to a pure motor subtype of GBS (Hao et al., 2019). An outbreak of GBS in 2007 in Jilin province was found related to *C. jejuni* infection in summer days (Zhang et al., 2010). The *C. jejuni* was still the predominant organism in the patients with GBS, but the frequency was much lower (Hao et al., 2019). In our study, the patients with the axonal subtype have a higher proportion of gastroenteritis than the patients with other subtypes but URTI, which was still the most common preceding event. Some patients with *C. jejuni* infection may have symptoms of URTI; thus, we need a perspective study for more information. In South China, the patients have a higher percentage of URTI than the patients in more northern regions. That indicated that the preceding infection may differ among regions, partially because the climate and eating habits are different in northern and southern regions.

Our study still has some limitations. First, as a retrospective study, some missing information is inevitable; long-term follow-up was unavailable, so we need a prospective study for further study. Second, the axonal subtype of GBS needs a series of electrophysiological examinations for diagnosis; otherwise, the proportion of axonal GBS may be underestimated. In our study, about 70% of the patients finished their NCS after 2 weeks from the onset, and we compare the NCS findings in different timing but found no difference in the ratio of the axonal type. Third, as a retrospective study, extracting bias was anticipated; we train our members strictly and use a unified standard to analyze the NCS data, and uncertain results were discussed to make a conclusion.

## Conclusion

There were significant differences among regions in China. Seasonal variation and preceding events’ differences lead to the heterogeneity of the patients with GBS in subtypes and a clinical course. A prospective study is needed for more accurate results.

## Data Availability Statement

The raw data supporting the conclusions of this article will be made available by the authors, without undue reservation.

## Ethics Statement

The studies involving human participants were reviewed and approved by Ethics Committee of Remin Hospital of Wuhan university. Written informed consent from the participants’ legal guardian/next of kin was not required to participate in this study in accordance with the national legislation and the institutional requirements.

## Author Contributions

ZL designed the study and organized the database. JY, SL, and YL contributed to the data collection and organization. JY wrote the manuscript. All authors contributed to the article and approved the submitted version.

## Conflict of Interest

The authors declare that the research was conducted in the absence of any commercial or financial relationships that could be construed as a potential conflict of interest.

## Publisher’s Note

All claims expressed in this article are solely those of the authors and do not necessarily represent those of their affiliated organizations, or those of the publisher, the editors and the reviewers. Any product that may be evaluated in this article, or claim that may be made by its manufacturer, is not guaranteed or endorsed by the publisher.
