# Supplementary material for: Regional Differences of Guillain-Barré Syndrome in China: From South to North
Source: Front Aging Neurosci. 2022 Feb 1;14:831890. doi: 10.3389/fnagi.2022.831890 (PMC8845027; doi:10.3389/fnagi.2022.831890)
Supplement: Supplementary file 1 [file Table_1.pdf]

Supplemental table 1. Subtype distribution of different seasons

	Demyelinating	Axonal	Inexcitable	Equivocal	Normal	P value
Winter-spring	109(38)	60(21)	2(1)	59(21)	55(19)	0.156
Summer-autumn	134(45)	75(25)	2(1)	46(15)	44(15)	
