# Supplementary material for: Regional Differences of Guillain-Barré Syndrome in China: From South to North
Source: Front Aging Neurosci. 2022 Feb 1;14:831890. doi: 10.3389/fnagi.2022.831890 (PMC8845027; doi:10.3389/fnagi.2022.831890)
Supplement: Supplementary file 2 [file Table_2.pdf]

Supplement Table 2. Hospitals in 14 provinces in our study

Provinces	Hospitals
Hubei	Renmin Hospital of Wuhan University
	Zhongnan Hospital of Wuhan University
	Union Hospital, Tongji Medical College, Huazhong University of Science and Technology
	Tongji Hospital, Tongji Medical College, Huazhong University of Science and Technology,
Henan	Henan provincial people’s Hospital
	The First Affliated Hospital of Zhengzhou University
	The Fifth Affliated Hospital of Zhengzhou University
Hunan	Xiangya Hospital of Central South University
	The Third Xiangya Hospital of Central South University
Shanghai	Shanghai Jiaotong University Affiliated First People's Hospital
Zhejiang	The Second Affiliated Hospital of Zhejiang University
	First Affiliated Hospital of College of Medicine, Zhejiang University,
	The First Affiliated Hospital of Wenzhou Medical University
Fujian	Fujian Provincial Hospital
	Fujian Medical University Union Hospital,
Jiangsu	Northern Jiangsu People's Hospital
Jiangxi	Jiangxi Provincial People's Hospital
Chongqing	The Second Affiliated Hospital of Chongqing Medical University
Sichuan	Sichuan Academy of Medical Sciences & Sichuan Provincial People's Hospital
	Affiliated Hospital of North Sichuan Medical College
Guizhou	Guizhou Provincial People's Hospital
Guangxi	The First Affiliated Hospital of Guangxi Medical University
Hainan	Hainan General Hospital
Guangdong	The First Affiliated Hospital of Shenzhen University
